# Supplementary material for: Crispant analysis in zebrafish as a tool for rapid functional screening of disease-causing genes for bone fragility
Source: eLife. 2025 Jan 16;13:RP100060. doi: 10.7554/eLife.100060 (PMC11737869; doi:10.7554/eLife.100060)
Supplement: Supplementary file 1. — (a) Overview of selected genes for crispant analysis, with reported mutations and/or polymorphisms associated with skeletal and non-skeletal phenotypes in human, mice and zebrafish. The conservation between human and zebrafish is reported in the last column. The first four genes are associated with the pathogenesis of osteoporosis, while the last six are linked to osteogenesis imperfecta. (b) Assessment of off-target effects in crispants. Genes that are possibly targeted by the selected crRNA for each of the crispants are called ‘off-target genes’ in this table (mm=number of mismatches). The top 3 ranked off-target effects, selected based on a high CFD (cutting frequency determination) score, are listed, together with their chromosomal position, forward and reverse primer for amplification, the CFD (cutting frequency determination) score and the off-target percentage, based on NGS analysis of a pool of DNA of 1-day old crispants (n=10). (c) Crispant genotyping. Crispant genes with crRNA sequence, forward and reverse primers and assay specifications for Next-generation sequencing (NGS) are listed in this table. The first four genes are associated with the pathogenesis of osteoporosis, while the last six are linked to osteogenesis imperfecta. Primers are designed using Primer3 (https://primer3.ut.ee). (d) qPCR primers. Skeletal marker genes and reference genes with forward and reverse primers are listed in this table. Primers are designed using NCBI PrimerBlast (https://www.ncbi.nlm.nih.gov/tools/primer-blast). [file elife-100060-supp1.docx]

**Supplementary Table 1a:** *Overview of selected genes for crispant analysis, with reported mutations and/or polymorphisms associated with skeletal and non-skeletal phenotypes in human, mice and zebrafish. The conservation between human and zebrafish is reported in the last column.* *The first four genes are associated with the pathogenesis of osteoporosis, while the last six are linked to osteogenesis imperfecta.*

|  | Human | | |  | Mouse | | | |  | Zebrafish | | |  |
| --- | --- | --- | --- | --- | --- | --- | --- | --- | --- | --- | --- | --- | --- |
|  | Variant | Phenotypic features | Skeletal abnormalities |  | Mutation/model | Phenotypic features | Skeletal abnormalities | |  | Mutation/model | Phenotypic features | Skeletal abnormalities | Conservation |
| *ALDH7A1* | c.1279G>C [1] | Pyridoxine-Dependent Epilepsy (PDE) phenotype | n/a |  | *Aldh7a1* KO mice [2] | Early death | | n/a |  | *aldh7a1*-null zebrafish [3] | PDE phenotype with premature death at 14 dpf | n/a | 82% |
|  |  |  |  |  |  |  | |  |  |  |  |  |  |
|  | Polymorphisms [4] [5][6] | n/a | Associated with bone mineral density |  | / | / | | / |  | *aldh7a1* Morpholino knockdown [7] | n/a | Defects in cartilage and pectoral fin development |  |
| *DAAM2* | Missense mutation [8]  Polymorphisms [9][10][11] | Nephrotic syndrome  n/a | n/a  Associated with bone mineral density variation |  | Hypomorphic *Daam2* allele [9]  / | n/a  / | | Reduced bone strength and increased cortical bone porosity  / |  | /  / | /  / | /  / | 72% |
| *ESR1* | Polymorphisms [12][13][14][15][16][17][18] | Associated with different diseases, including breast cancer and cardiovascular risk | Associated with bone mineral density variation |  | *ERα* KO mice [19] | n/a | | Decrease in cortical bone mineral density and increase in trabecular bone mineral density |  | Loss-of-function zebrafish [20] | Viable and overall normal  Role of *esr1* in regulation of heart rate is discovered | n/a | 47% |
| *SOST* | Nonsense mutation [21]  Polymorphisms [12][15][16][17][22] | Sclerosteosis  n/a | Progressive bone thickening and increased BMD  Associated with bone mineral density variation |  | *Sost* KO mice [23]  / | n/a  / | | Increase in bone volume, BMD and osteoblast surface area  / |  | n/a  / | n/a  / | n/a  / | 50% |
| *CREB3L1* | c.934_936delAAG [24]  c.1284C-A [25]  c.911C-T [26] | n/a | Osteogenesis Imperfecta type XVI |  | *Oasis* KO mice [27] | n/a | | Decreased bone density, delayed osteoblast maturation resulting in severe osteopenia |  | *creb3l1*^ΔbZIP/ΔbZIP^ zebrafish [28] | n/a | Upregulation of *creb3l1* expression | 59% |
| *IFITM5* | c. 14C>T [29]  c.119C>T [30]  c.119C>G [31]  / | n/a  / | Osteogenesis Imperfecta type V  / |  | c. 14C>T [32]  *Ifitm5* KO mice [33] | n/a  n/a | | Severe skeletal defects: fractures and early lethality Abnormalities in mineralization  No skeletal abnormalities |  | n/a  / | n/a  / | n/a  / | 39% |
| *MBTPS2* | Missense mutation [34][35]  / | X-linked Olmsted syndrome and IFAP Syndrome 1, with or without BRESHECK Syndrome  / | X-linked Osteogenesis Imperfecta  / |  | *Mbtps2* Knock-in (N455S) [36]  *Mbtps2* KO mice [36] | n/a  n/a | | Embryonic lethality in hemizygous male mice, early osteoarthritis in heterozygous female mice  Embryonic lethality in hemizygous male mice, early osteoarthritis in heterozygous female mice |  | n/a  / | n/a  / | n/a  / | 60% |
| *SEC24D* | Nonsense variants [37][38]  Missense variants [37][38][39]  Frameshift variants [40][41][39] | n/a | Autosomal recessive Osteogenesis Imperfecta with a Cole-Carpenter syndrome-like phenotype |  | *Sec24d* KO mice [42] | Embryonic lethality | | Embryonic lethality |  | *sec24d* KO zebrafish (*Bulldog*) [43] |  | Abnormalities in pectoral fin and head skeleton | 65% |
| *SERPINF1* | c.696C>G [44]  c.324_325dupCT [44]  c.1132C>T [44]  c.1118_1119del [45]  c.1-4796dupT [45]  c.653delT [45] | n/a | Osteogenesis Imperfecta type VI |  | PEDF KO mice [46] | Retinal malformations | | Mild reduction in trabecular bone volume and accumulation of unmineralized bone matrix: increased bone fragility |  | n/a | n/a | n/a | 39% |
| *SPARC* | c.497G>A [47]  c.787G>A [47] | n/a | Osteogenesis Imperfecta type XVII |  | *Sparc* null mice [48] | Abnormal eye phenotype | | Decrease in BMD, bone mineral content and increase in bone fragility |  | n/a | n/a | n/a | 77% |

**Supplementary Table 1b: Assessment of off-target effects in crispants.** *Genes that are possibly targeted by the selected crRNA for each of the crispants are called ‘off-target genes’ in this table (mm=number of mismatches). The top 3 ranked off-target effects, selected based on a high CFD (cutting frequency determination) score, are listed, together with their chromosomal position, forward and reverse primer for amplification, the CFD (cutting frequency determination) score and the off-target percentage, based on NGS analysis of a pool of DNA of 1-day old crispants (n=10).*

**Supplementary Table 1c: Crispant genotyping.** *Crispant genes with crRNA sequence, forward and reverse primers and assay specifications for Next-generation sequencing (NGS) are listed in this table. The first four genes are associated with the pathogenesis of osteoporosis, while the last six are linked to osteogenesis imperfecta. Primers are designed using Primer3 (*[*https://primer3.ut.ee*](https://primer3.ut.ee)*).*

| **Gene** | **crRNA** | **Forward primer** | **Reverse primer** | **Assay** |
| --- | --- | --- | --- | --- |
| *aldh7a1* | AATTGTTCGACAGATTGGAG | CCTATTTGCTTAGTTAAATTCAGGTGG | CATCCACATACTCCTGCACC | FORD58 |
| *daam2* | GATCTATTGCAGTAAGAAGA | AGTGTGGCTCAGTTGATGTGT | ACATGTCTTTGGATTGCCCCT | FORD60 |
| *esr1* | CAGCTCCTCACACAGGCCCA | TGTTTCCATGGTGATGTCTGG | TGTTAGTAAATACATCAACTCAAAGACC | FORD60 |
| *sost* | GTTTGATCACAATATGCCAG | GTGCCGTCCATCAACAGC | GGTGAACTTGAAATGAACCGC | FORD60 |
| *creb3l1* | ACACAGTTACTCTCTCAGCG | TGTATCCGTTTGCTACCATACC | TGTTTGTGTGGATGGATGGC | FORD60 |
| *ifitm5* | AGCGCAGGAATGCTCAGACA | TCCATCAAGGCTCGAGATCAG | TGAGAAATTCACACAGCCAAGG | FORD60 |
| *mbtps2* | TTCCATATCAAGTGGCACAC | TCTCCTTCTGTCTGTTTCACC | ACTCGGATGGAAAGCAAAGC | FORD60 |
| *sec24d* | GCCTATGGATCTCCAACACA | AAATTGAAACTACAGTGCATAATGG | GGGTCCATTGTTCATCTGAGG | FORD60 |
| *serpinf1* | GTGTTACGAGGAAGAGGGGA | TCTCTCTGTCATTCCGCTGG | GTAAATCTGCTTCTCGGCCC | FORD60 |
| *sparc* | CTGCCAGAGTCTTGCCAGCG | CTACAGCCCTTTCATTCCAGG | TGACCCAAACACATAACTATAAGC | FORD60 |

**Supplementary Table 1d: qPCR primers.** *Skeletal marker genes and reference genes with forward and reverse primers are listed in this table. Primers are designed using NCBI PrimerBlast (*[*https://www.ncbi.nlm.nih.gov/tools/primer-blast*](https://www.ncbi.nlm.nih.gov/tools/primer-blast/index.cgi?GROUP_TARGET=on)*).*

| **Gene** | **FWD primer** | **RVS primer** |
| --- | --- | --- |
| **Skeletal marker genes** | | |
| *runx2a* | GGAAGAGGAAAGAGCTTCAC | CGTCCACTGTGACCTTTATG |
| *sp7* | CTCTCCTCTCCCGCTTT | GTGTTTCCTCCTCCAGAATC |
| *sox9a* | TGGGAAAACTTTGGAGATTACTGA | AGTCGGGGTGATCTTTCTTGT |
| *sox9b* | GAAGATGGAGAGCAGACGCA | CCTGAGACTGACCGGAGTG |
| *bglap* | TCTTCCTGACTCCTCAGATAC | AGCCCTCTTCTGTCTCAT |
| *col1a1a* | TCTGGTGGCTTTGATGAG | GGGACCAGTAAATCCTGGG |
| *col1a1b* | CTTGCAGTGAGAGGACAA | GCTCGGGTTTCCATACAT |
| *col1a2* | TAACCCTGGTGCTAATGGTA | ACACCAGAATCTCCCTTCA |
| *col2a1a* | GAGAACCAGGCGATATTACA | CACCCTTAGCTCGTCTTTC |
| *col2a1b* | GTTGGTACAGCAGGATCTC | TCCAGGAACACCAGACT |
| **Reference genes** | | |
| *elfa* | GGAGACTGGTGTCCTCAA | GGTGCATCTCAACAGACTT |
| *bactin2* | ACGATGGATGGGAAGACA | AAATTGCCGCACTGGTT |

**References**

[1] Coughlin CR, Swanson MA, Spector E, Meeks NJL, Kronquist KE, Aslamy M, et al. The genotypic spectrum of ALDH7A1 mutations resulting in pyridoxine dependent epilepsy: A common epileptic encephalopathy. J Inherit Metab Dis 2019;42:353–61. https://doi.org/10.1002/jimd.12045.

[2] Al-Shekaili HH, Petkau TL, Pena I, Lengyell TC, Verhoeven-Duif NM, Ciapaite J, et al. A novel mouse model for pyridoxine-dependent epilepsy due to antiquitin deficiency. Hum Mol Genet 2020;29:3266–84. https://doi.org/10.1093/hmg/ddaa202.

[3] Pena IA, Roussel Y, Daniel K, Mongeon K, Johnstone D, Mendes HW, et al. Pyridoxine-dependent epilepsy in zebrafish caused by aldh7a1 deficiency. Genetics 2017;207:1501–18. https://doi.org/10.1534/genetics.117.300137.

[4] Guo Y, Tan LJ, Lei SF, Yang TL, Chen XD, Zhang F, et al. Genome-wide association study identifies ALDH7A1 as a novel susceptibility gene for osteoporosis. PLoS Genet 2010;6. https://doi.org/10.1371/journal.pgen.1000806.

[5] Zhu X, Bai W, Zheng H. Twelve years of GWAS discoveries for osteoporosis and related traits: advances, challenges and applications. Bone Res 2021;9. https://doi.org/10.1038/s41413-021-00143-3.

[6] Sadat-Ali M, Al-Turki RA, Al-Turki HA, Almohaya MS. Genetic influence on osteoporosis and fracture risk: Outcome of genome-wide association studies – A systematic review. Journal of Musculoskeletal Surgery and Research 2024;8. https://doi.org/10.25259/JMSR_220_2023.

[7] Babcock HE, Dutta S, Alur RP, Brocker C, Vasiliou V, Vitale S, et al. Aldh7a1 regulates eye and limb development in zebrafish. PLoS One 2014;9. https://doi.org/10.1371/journal.pone.0101782.

[8] Schneider R, Deutsch K, Hoeprich GJ, Marquez J, Hermle T, Braun DA, et al. DAAM2 Variants Cause Nephrotic Syndrome via Actin Dysregulation. Am J Hum Genet 2020;107:1113–28. https://doi.org/10.1016/j.ajhg.2020.11.008.

[9] Morris JA, Kemp JP, Youlten SE, Laurent L, Logan JG, Chai RC, et al. An atlas of genetic influences on osteoporosis in humans and mice. Nat Genet 2019;51:258–66. https://doi.org/10.1038/s41588-018-0302-x.

[10] Kichaev G, Bhatia G, Loh PR, Gazal S, Burch K, Freund MK, et al. Leveraging Polygenic Functional Enrichment to Improve GWAS Power. Am J Hum Genet 2019;104:65–75. https://doi.org/10.1016/j.ajhg.2018.11.008.

[11] Kim SK. Identification of 613 new loci associated with heel bone mineral density and a polygenic risk score for bone mineral density, osteoporosis and fracture. PLoS One 2018;13. https://doi.org/10.1371/journal.pone.0200785.

[12] Brent Richards J, Kavvoura FK, Rivadeneira F, Styrkársdóttir U, Estrada K, Halldórsson B V, et al. Collaborative Meta-analysis: Associations of 150 Candidate Genes With Osteoporosis and Osteoporotic Fracture. n.d.

[13] Styrkarsdottir U, Halldorsson B V., Gretarsdottir S, Gudbjartsson DF, Walters GB, Ingvarsson T, et al. Multiple Genetic Loci for Bone Mineral Density and Fractures. New England Journal of Medicine 2008;358:2355–65. https://doi.org/10.1056/nejmoa0801197.

[14] Rivadeneira F, Styrkársdottir U, Estrada K, Halldórsson B V., Hsu YH, Richards JB, et al. Twenty bone-mineral-density loci identified by large-scale meta-analysis of genome-wide association studies. Nat Genet 2009;41:1199–206. https://doi.org/10.1038/ng.446.

[15] He D, Liu H, Wei W, Zhao Y, Cai Q, Shi S, et al. A longitudinal genome-wide association study of bone mineral density mean and variability in the UK Biobank. Osteoporosis International 2023;34:1907–16. https://doi.org/10.1007/s00198-023-06852-1.

[16] Surakka I, Fritsche LG, Zhou W, Backman J, Kosmicki JA, Lu H, et al. MEPE loss-of-function variant associates with decreased bone mineral density and increased fracture risk. Nat Commun 2020;11. https://doi.org/10.1038/s41467-020-17315-0.

[17] Medina-Gomez C, Kemp JP, Trajanoska K, Luan J, Chesi A, Ahluwalia TS, et al. Life-Course Genome-wide Association Study Meta-analysis of Total Body BMD and Assessment of Age-Specific Effects. Am J Hum Genet 2018;102:88–102. https://doi.org/10.1016/j.ajhg.2017.12.005.

[18] Comuzzie AG, Cole SA, Laston SL, Voruganti VS, Haack K, Gibbs RA, et al. Novel Genetic Loci Identified for the Pathophysiology of Childhood Obesity in the Hispanic Population. PLoS One 2012;7. https://doi.org/10.1371/journal.pone.0051954.

[19] Melville KM, Kelly NH, Khan SA, Schimenti JC, Ross FP, Main RP, et al. Female mice lacking estrogen receptor-alpha in osteoblasts have compromised bone mass and strength. Journal of Bone and Mineral Research 2014;29:370–9. https://doi.org/10.1002/jbmr.2082.

[20] Romano SN, Edwards HE, Souder JP, Ryan KJ, Cui X, Gorelick DA. G protein-coupled estrogen receptor regulates embryonic heart rate in zebrafish. PLoS Genet 2017;13. https://doi.org/10.1371/journal.pgen.1007069.

[21] Brunkow ME, Gardner JC, Ness J Van, Paeper BW, Kovacevich BR, Proll S, et al. Bone Dysplasia Sclerosteosis Results from Loss of the SOST Gene Product, a Novel Cystine Knot-Containing Protein. vol. 68. 2001.

[22] Styrkarsdottir U, Halldorsson B V, Gretarsdottir S, Gudbjartsson DF, Walters GB, Ingvarsson T, et al. New sequence variants associated with bone mineral density. Nat Genet 2009;41:15—17. https://doi.org/10.1038/ng.284.

[23] Li X, Ominsky MS, Niu QT, Sun N, Daugherty B, D’Agostin D, et al. Targeted deletion of the sclerostin gene in mice results in increased bone formation and bone strength. Journal of Bone and Mineral Research 2008;23:860–9. https://doi.org/10.1359/jbmr.080216.

[24] Keller RB, Tran TT, Pyott SM, Pepin MG, Savarirayan R, McGillivray G, et al. Monoallelic and biallelic CREB3L1 variant causes mild and severe osteogenesis imperfecta, respectively. Genetics in Medicine 2018;20:411–9. https://doi.org/10.1038/gim.2017.115.

[25] Lindahl K, Åström E, Dragomir A, Symoens S, Coucke P, Larsson S, et al. Homozygosity for CREB3L1 premature stop codon in first case of recessive osteogenesis imperfecta associated with OASIS-deficiency to survive infancy. Bone 2018;114:268–77. https://doi.org/10.1016/j.bone.2018.06.019.

[26] Guillemyn B, Kayserili H, Demuynck L, Sips P, De Paepe A, Syx D, et al. A homozygous pathogenic missense variant broadens the phenotypic and mutational spectrum of CREB3L1-related osteogenesis imperfecta. Hum Mol Genet 2019;28:1801–9. https://doi.org/10.1093/hmg/ddz017.

[27] Sekiya H, Murakami T, Saito A, Hino SI, Tsumagari K, Ochiai K, et al. Effects of the bisphosphonate risedronate on osteopenia in OASIS-deficient mice. J Bone Miner Metab 2010;28:384–94. https://doi.org/10.1007/s00774-009-0142-y.

[28] Vanwinkle PE, Lee E, Wynn B, Nawara TJ, Thomas H, Parant J, et al. Disruption of the creb3l1 gene causes defects in caudal fin regeneration and patterning in zebrafish Danio rerio. n.d.

[29] Semler O, Garbes L, Keupp K, Swan D, Zimmermann K, Becker J, et al. A mutation in the 5′-UTR of IFITM5 creates an in-frame start codon and causes autosomal-dominant osteogenesis imperfecta type v with hyperplastic callus. Am J Hum Genet 2012;91:349–57. https://doi.org/10.1016/j.ajhg.2012.06.011.

[30] Guillén-Navarro E, Ballesta-Martínez MJ, Valencia M, Bueno AM, Martinez-Glez V, López-González V, et al. Two mutations in IFITM5 causing distinct forms of osteogenesis imperfecta. Am J Med Genet A 2014;164:1136–42. https://doi.org/10.1002/ajmg.a.36409.

[31] Lim JY, Bhatia NS, Vasanwala RF, Chay PL, Lim KBL, Khoo PC, et al. A novel Ser40Trp variant in IFITM5 in a family with osteogenesis imperfecta and review of the literature. Clin Dysmorphol 2019;28:118–23. https://doi.org/10.1097/MCD.0000000000000279.

[32] Lietman CD, Marom R, Munivez E, Bertin TK, Jiang MM, Chen Y, et al. A transgenic mouse model of OI type V supports a neomorphic mechanism of the IFITM5 mutation. Journal of Bone and Mineral Research 2015;30:498–507. https://doi.org/10.1002/jbmr.2363.

[33] Hanagata N, Li X, Morita H, Takemura T, Li J, Minowa T. Characterization of the osteoblast-specific transmembrane protein IFITM5 and analysis of IFITM5-deficient mice. J Bone Miner Metab 2011;29:279–90. https://doi.org/10.1007/s00774-010-0221-0.

[34] Oeffner F, Fischer G, Happle R, König A, Betz RC, Bornholdt D, et al. IFAP Syndrome Is Caused by Deficiency in MBTPS2, an Intramembrane Zinc Metalloprotease Essential for Cholesterol Homeostasis and ER Stress Response. Am J Hum Genet 2009;84:459–67. https://doi.org/10.1016/j.ajhg.2009.03.014.

[35] Lindert U, Cabral WA, Ausavarat S, Tongkobpetch S, Ludin K, Barnes AM, et al. MBTPS2 mutations cause defective regulated intramembrane proteolysis in X-linked osteogenesis imperfecta. Nat Commun 2016;7. https://doi.org/10.1038/ncomms11920.

[36] Danyukova T, Alimy AR, Velho RV, Yorgan TA, Di Lorenzo G, von Kroge S, et al. Mice heterozygous for an osteogenesis imperfecta-linked MBTPS2 variant display a compromised subchondral osteocyte lacunocanalicular network associated with abnormal articular cartilage. Bone 2023;177. https://doi.org/10.1016/j.bone.2023.116927.

[37] Garbes L, Kim K, Rieß A, Hoyer-Kuhn H, Beleggia F, Bevot A, et al. Mutations in SEC24D, encoding a component of the COPII machinery, cause a syndromic form of osteogenesis imperfecta. Am J Hum Genet 2015;96:432–9. https://doi.org/10.1016/j.ajhg.2015.01.002.

[38] Takeyari S, Kubota T, Miyata K, Yamamoto K, Nakayama H, Yamamoto K, et al. Japanese patient with Cole-carpenter syndrome with compound heterozygous variants of SEC24D. Am J Med Genet A 2018;176:2882–6. https://doi.org/10.1002/ajmg.a.40643.

[39] Li S, Cao Y, Wang H, Li L, Ren X, Mi H, et al. Genotypic and Phenotypic Analysis in Chinese Cohort With Autosomal Recessive Osteogenesis Imperfecta. Front Genet 2020;11. https://doi.org/10.3389/fgene.2020.00984.

[40] Moosa S, Chung BHY, Tung JYL, Altmüller J, Thiele H, Nürnberg P, et al. Mutations in SEC24D cause autosomal recessive osteogenesis imperfecta. Clin Genet 2016;89:517–9. https://doi.org/10.1111/cge.12678.

[41] Mooatt P, Marulanda J, Wu D. Case report: Clinical manifestations and genotype analysis of a child with PTPN11 and SEC24D mutations. n.d.

[42] Baines AC, Adams EJ, Zhang B, Ginsburg D. Disruption of the Sec24d Gene Results in Early Embryonic Lethality in the Mouse. PLoS One 2013;8. https://doi.org/10.1371/journal.pone.0061114.

[43] Sarmah S, Barrallo-Gimeno A, Melville DB, Topczewski J, Solnica-Krezel L, Knapik EW. Sec24D-dependent transport of extracellular matrix proteins is required for zebrafish skeletal morphogenesis. PLoS One 2010;5. https://doi.org/10.1371/journal.pone.0010367.

[44] Becker J, Semler O, Gilissen C, Li Y, Bolz HJ, Giunta C, et al. Exome sequencing identifies truncating mutations in human SERPINF1 in autosomal-recessive osteogenesis imperfecta. Am J Hum Genet 2011;88:362–71. https://doi.org/10.1016/j.ajhg.2011.01.015.

[45] Shaheen R, Alazami AM, Alshammari MJ, Faqeih E, Alhashmi N, Mousa N, et al. Study of autosomal recessive osteogenesis imperfecta in Arabia reveals a novel locus defined by TMEM38B mutation n.d. https://doi.org/10.1136/jmedgenet-2012.

[46] Dixit S, Polato F, Samardzija M, Abu-Asab M, Grimm C, Crawford SE, et al. PEDF deficiency increases the susceptibility of rd10 mice to retinal degeneration. Exp Eye Res 2020;198. https://doi.org/10.1016/j.exer.2020.108121.

[47] Mendoza-Londono R, Fahiminiya S, Majewski J, Tétreault M, Nadaf J, Kannu P, et al. Recessive Osteogenesis Imperfecta Caused by Missense Mutations in SPARC. Am J Hum Genet 2015;96:979–85. https://doi.org/10.1016/j.ajhg.2015.04.021.

[48] Gilmour DT, Lyon GJ, Carlton MBL, Sanes JR, Cunningham JM, Anderson JR, et al. Mice deficient for the secreted glycoprotein SPARC/osteonectin/BM40 develop normally but show severe age-onset cataract formation and disruption of the lens. vol. 17. 1998.
